# Rice Bodies Accompanied by Tenosynovitis of the Wrist: A Case Report and Literature Review

**DOI:** 10.7759/cureus.29682

**Published:** 2022-09-28

**Authors:** Maher Ghandour, Tanios Dagher, Anthony Tannous, Nancy Zeaiter, Sami Salem

**Affiliations:** 1 Orthopaedics and Traumatology, Heidelberg University Hospital, Heidelberg, DEU; 2 Orthopaedics and Traumatology, Lebanese Hospital Geitaoui, Beirut, LBN; 3 Orthopaedics and Traumatology, Lebanese University Faculty of Medicine, Beirut, LBN; 4 Plastic and Reconstructive Surgery, Lebanese University, Beirut, LBN

**Keywords:** mri imaging, tenosynovitis, wrist, rheumatoid arthriitis, rice bodies

## Abstract

Rice bodies, a rare finding in clinical practice, are commonly observed in the shoulders and knees of affected individuals. However, they can occur in the wrist as well. Herein, we report a case of a female presenting with painful swelling in the right wrist that lasted with a history of carpal tunnel syndrome, rheumatoid arthritis, and history of median nerve decompression two years ago. A potential diagnosis of infectious diseases and gout was excluded through negative cultures and laboratory findings. X-rays showed no significant findings; however, magnetic resonance imaging revealed findings suggestive of rice bodies that were confirmed by additional proton dense fat-saturated imaging. The mass was then removed by extensive debridement and sent for pathological assessment, which showed multiple nodules containing fibrin and polymorphonuclear cells. The patient did not experience recurrence during the follow-up period. Rice bodies, although rare, can occur in the wrist, and this imposes several challenges associated with their diagnostic and management protocols.

## Introduction

Rice bodies are rarely observed in clinical practice; however, they are associated with several diagnostic and treatment challenges. For instance, their occurrence has been reported to be closely associated with several rheumatic and infectious diseases, and multiple hypotheses have been proposed regarding their origin. Although they are commonly located in big joints like the shoulders and knees, rice bodies can also occur in the wrist [1]. Rice bodies are commonly associated with a chronic microinflammation of the synovium either in the context of rheumatoid arthritis or chronic infections. Available evidence highlights the occurrence of synovial hypertrophy with subsequent infarction as a cause for the occurrence of rice bodies [2]. However, the exact etiology is still not yet clearly understood. The diagnosis of rice bodies can be reached through proper history, thorough physical examination, and intensive radiological assessment through X-rays, computed tomography (CT), and magnetic resonance imaging (MRI), which ought to prompt orthopedic surgeons to rule out infection in clinical settings. Herein, we report a case of a female presenting with a swelling in the right wrist suggestive of rice bodies.

## Case presentation

**Figure 1:**
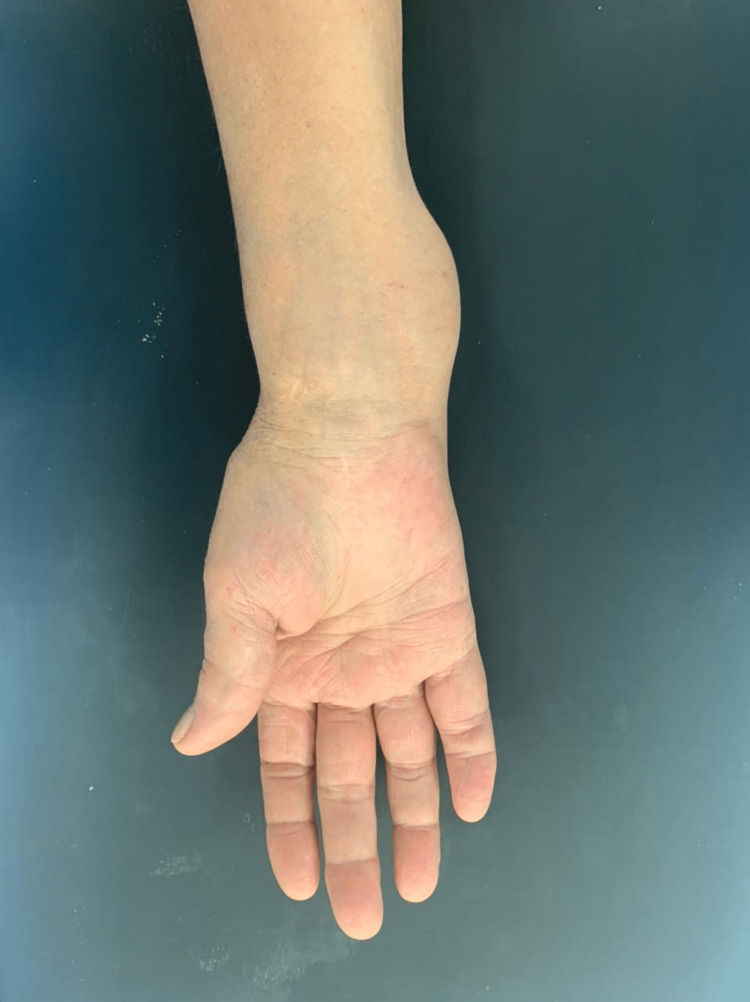
Swelling at the volar aspect of the right wrist upon presentation

A 73-year-old female patient presented to the orthopedic surgery department (Lebanese Hospital Geitaoui, Beirut) with median nerve palsy and an edematous right wrist with a circumferential mass located in this area. The patient complained of pain at the swelling site for three months, and she reported no prior injury to the wrist or pain before the swelling had occurred. Noteworthy, an informed consent was taken from the patient prior to the conduct of this research. The patient had no prior medical or surgical history except for a history of carpal tunnel syndrome, rheumatoid arthritis (five years prior to the presentation) on hydroxychloroquine medication, and right median nerve decompression two years prior to the current presentation. Physical examination of the right wrist and forearm was carried out, revealing an extensive volar swelling extending from the distal end of the forearm to the wrist (Figure 1). The swelling was not red; however, tenderness was evident. Tinel's and Phalen's tests for median nerve compression were positive. Laboratory investigations in terms of complete blood count and erythrocyte sedimentation rate were normal; however, there was a slight elevation in the C-reactive protein level.

**Figure 2:**
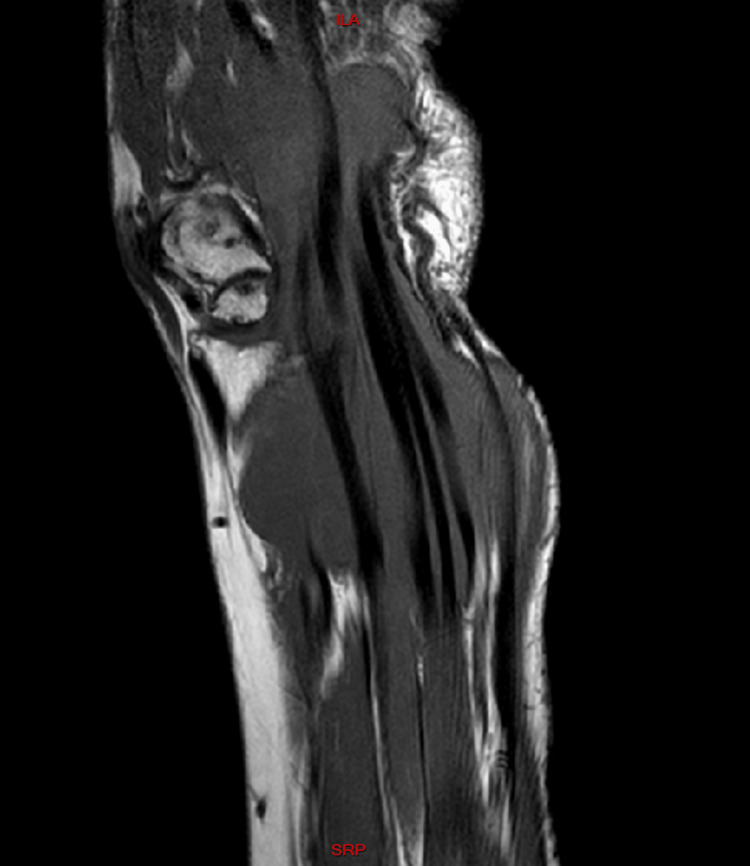
Coronal T1 image showing an isointense mass measuring 12x15 cm, extending from the distal forearm passing through the carpal tunnel and reaching the proximal palmar surface of the hand, surrounding the flexor tendons

**Figure 3:**
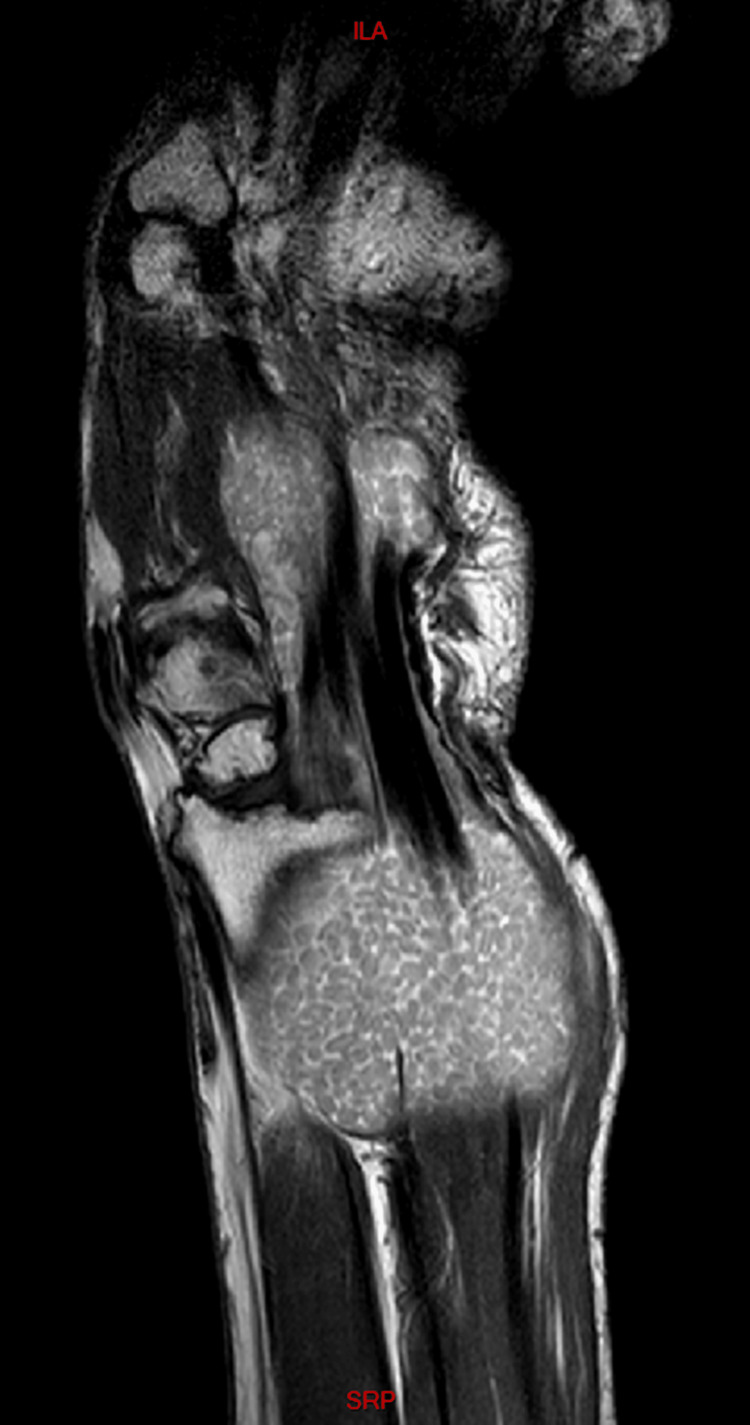
Coronal T2 weighted image showing multiple small nodular structures that appear isointense to muscles inside a hyperintense T2 mass involving the flexor tendons sheath, suggestive of tenosynovitis with rice body formation

**Figure 4:**
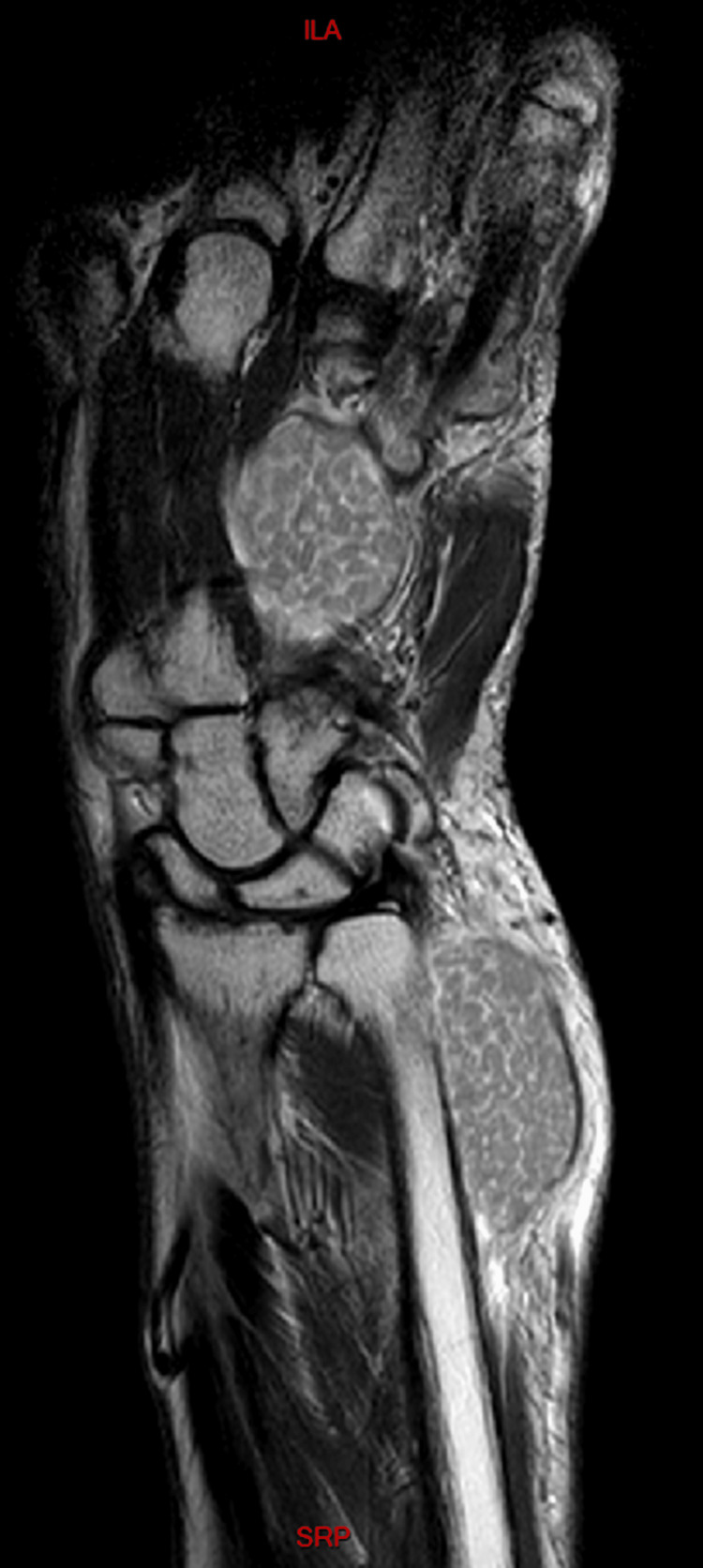
Rice body appearance extending from the distal aspect of the anterior compartment of the forearm to the level of the metacarpal bones

**Figure 5:**
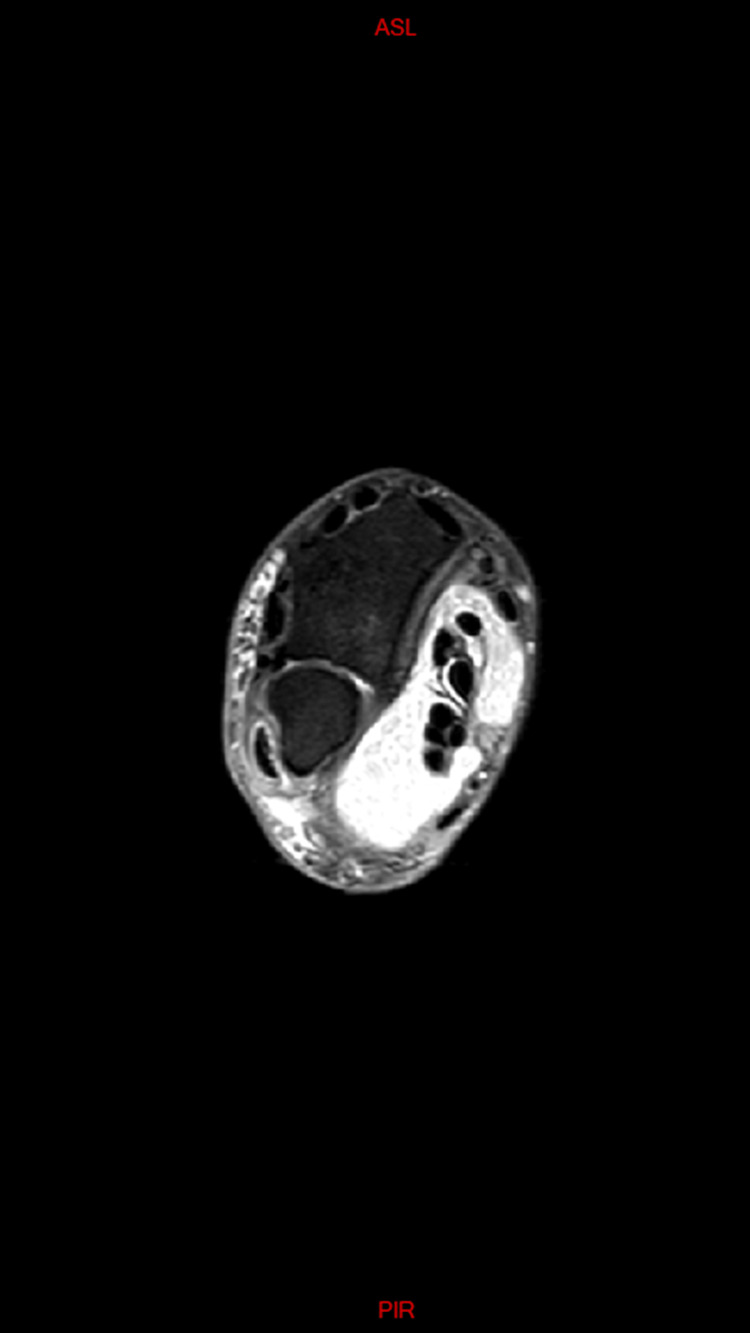
Axial PD Fat Sat weighted image at the level of the distal forearm reveals distention of the palmar bursae by complex material (rice bodies) against a background of fluid signal intensity PD, proton density; Fat Sat, fat saturated

**Figure 6:**
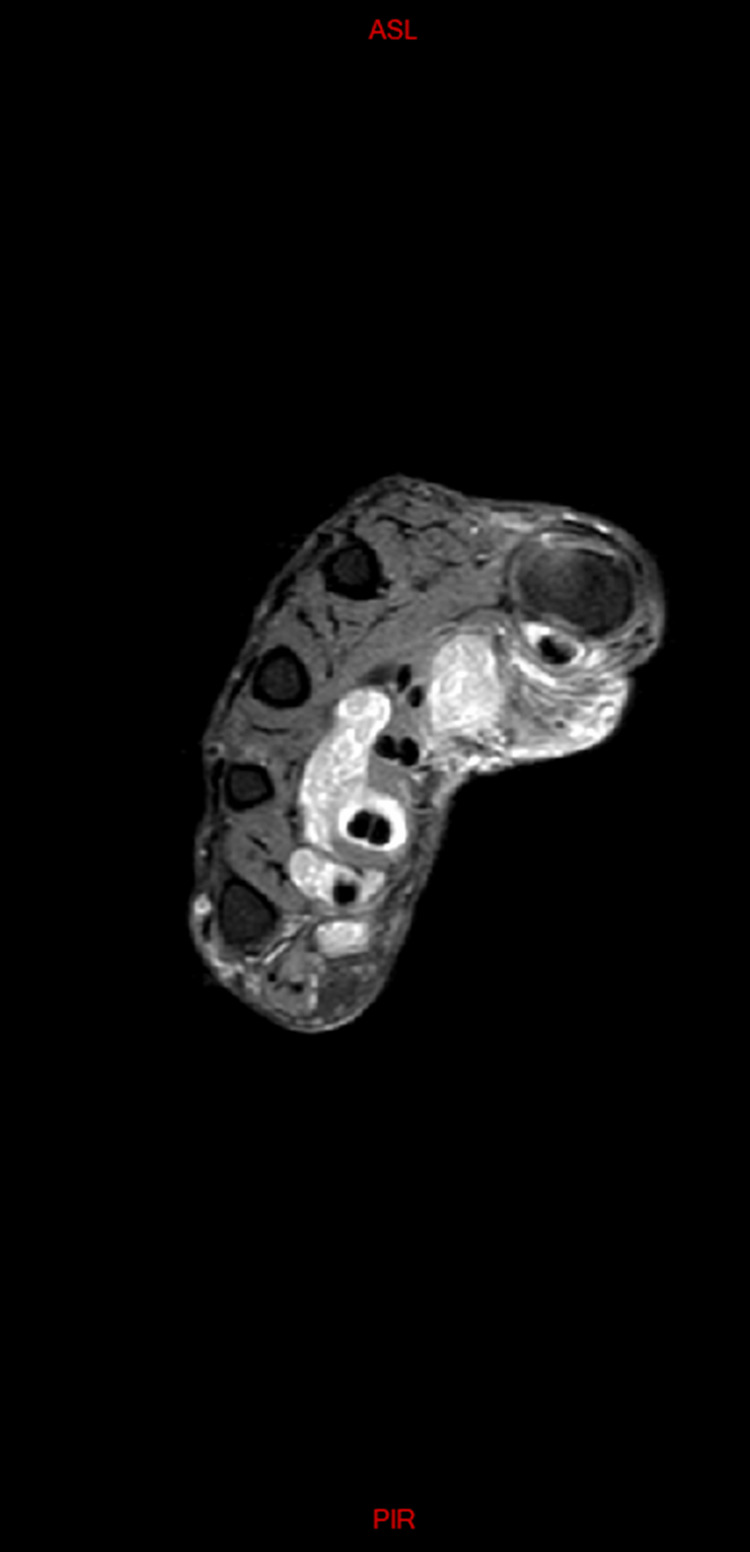
Axial PD Fat Sat weighted image at the level of the metacarpal heads showing extension of synovitis into the digital flexor tendon sheath of the thumb, long, ring and little fingers PD, proton density; Fat Sat, fat saturated

The initial differential diagnosis included the following possibilities: ganglion cyst, synovial cyst or synovitis, or giant cell tumor. We also put into consideration other etiologies like gout, synovial chondromatosis or abscesses. Cultures revealed no growth of germs of any kind, including tuberculosis. Also given the chronicity of the presenting sign, the possibility of gout was very dim. Radiological assessment showed normal X-rays of the right wrist; however, MRI images revealed a 12x15 cm isointense mass, extending from the distal forearm to the palmar surface of the hand (surrounding the flexor tendons) while passing through the carpal tunnel (Figure 2). Within this mass, multiple small nodular hypointense-to-isointense structures were observed inside a hyperintense T2 mass that involved the flexor tendons (Figure 3). This presentation was suggestive of tenosynovitis with rice body formation, as highlighted in the literature [1]. This rice body appearance was observed to extend from the distal aspect of the anterior compartment of the forearm to the level of metacarpals (Figure 4). Proton density (PD) fat saturated (Fat Sat) MRI analysis was performed for further confirmation. The findings revealed distention of the palmar bursae by complex bodies lying against a background of fluid signal intensity (Figure 5). In addition, synovitis was observed extending to the level of metacarpal heads and into the flexor tendon sheaths of the thumb, ring, long, and little fingers (Figure 6). According to these results, differential diagnosis other than tenosynovitis with rice body formation was rejected.

**Figure 7:**
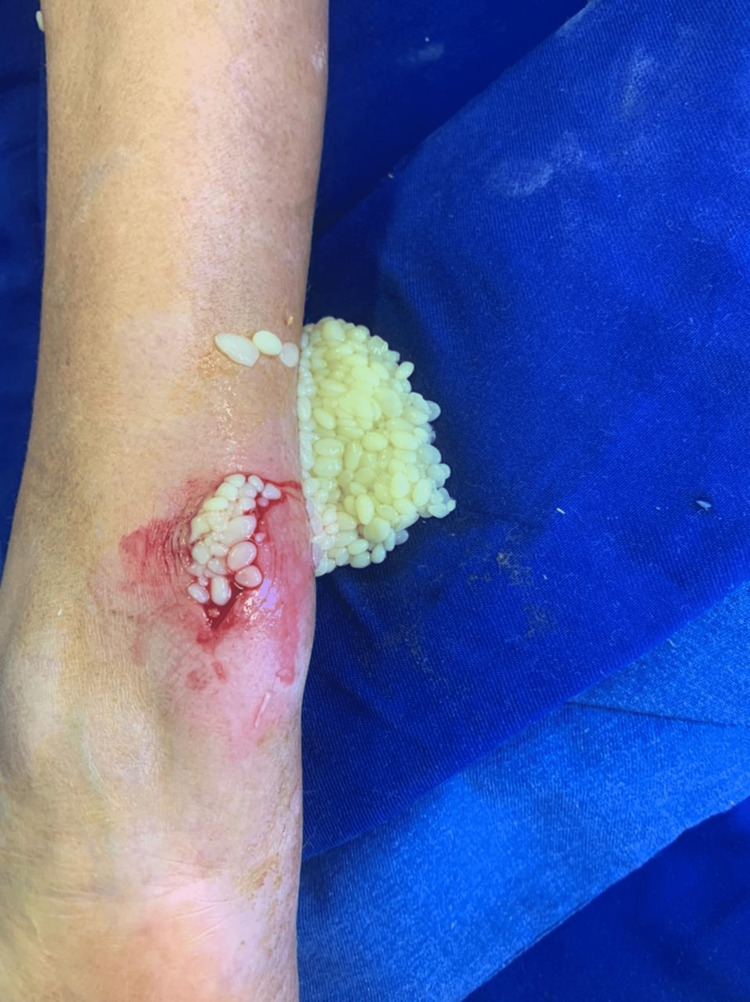
Rice bodies extracted during the operation

**Figure 8:**
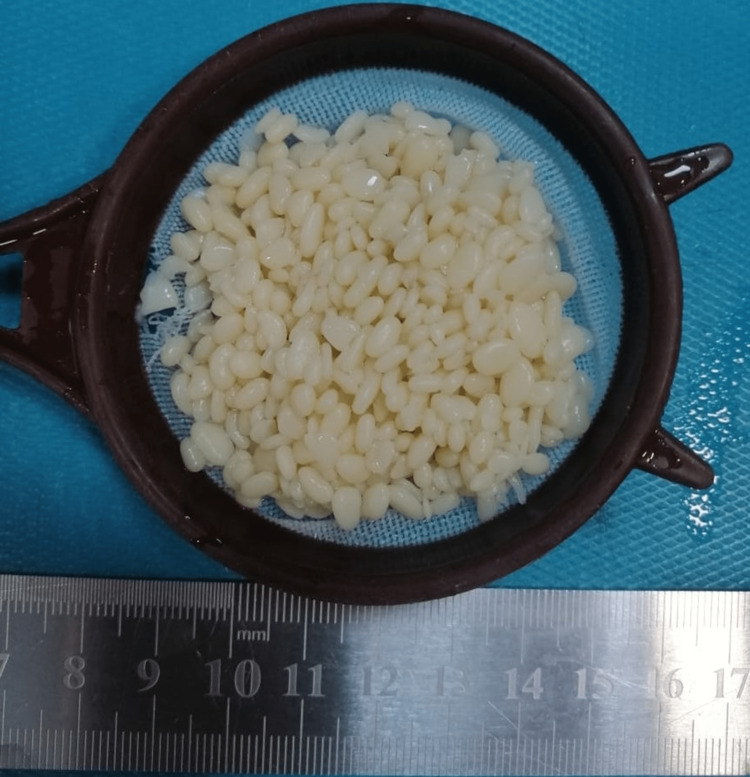
Multiple yellowish nodules, with a smooth surface, of different sizes ranging from 0.4 to 1 cm

**Figure 9:**
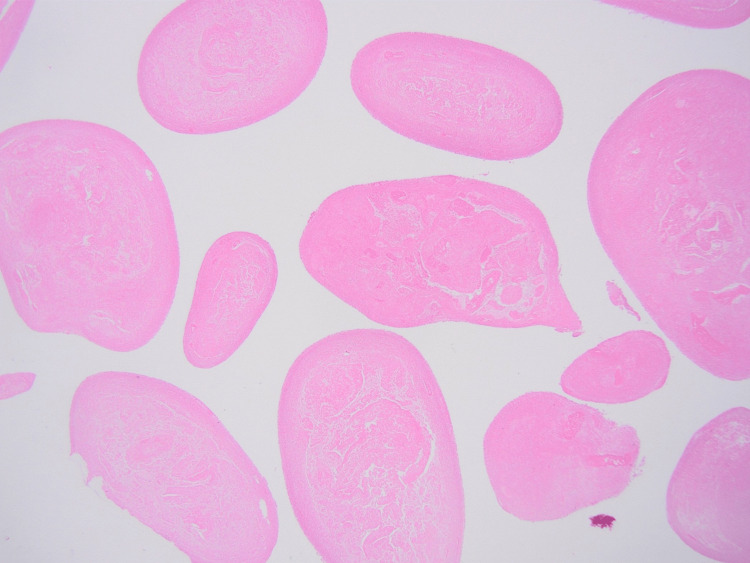
Hematoxylin and eosin stain showing rounded clusters of fibrin containing polymorphonuclear cells and karyorrhexic debris

**Figure 10:**
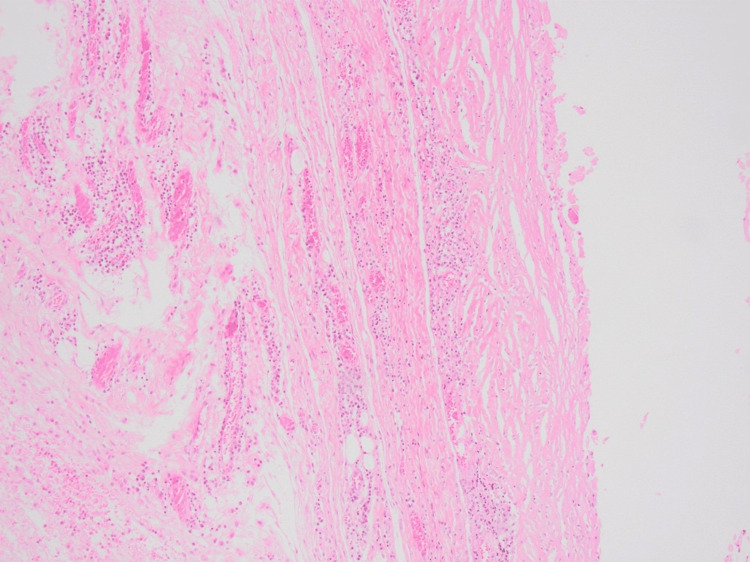
Hematoxylin and eosin stain showing a thickened and fibrous synovium containing an abundant inflammatory infiltrate

Following a thorough assessment, the decision to remove the rice bodies was reached. Chest X-ray before the procedure was done, and it was unremarkable. A written informed consent was taken from the patient before the operation. An extensive debridement procedure was carried out, where an incision was made over the mass, and a surgical spoon was used to extract the mass in addition to the applied pressure from the sides of the mass (to force the mass of rice bodies out). The extracted rice bodies are shown in Figure 7. At the end, washing was done, and through suction and dissection, we ensured that no rice bodies were left behind. Afterward, the collected sample was sent to the pathology department for further analysis. The gross examination of the removed mass revealed multiple yellowish nodules (0.4-1.0 cm in size) with a smooth surface (Figure 8). The microscopic examination showed yellowish nodules clustered to fibrin containing polymorphonuclear cells and karyorrhexic debris (Figure 9). The remaining fragments showed a thickened and fibrous synovium rich in inflammatory cells (plasma cells and lymphoid aggregates); however, no granuloma formation was observed (Figure 10). Overall, the microscopic examination was consistent with a subacute and chronic synovitis associated with the presence of numerous “rice bodies” consistent with the patient’s history of rheumatoid arthritis. Ziehl-Neelsen staining for acid-fast microorganisms was also done on the tissue sample, and it turned out negative; thus, tuberculosis was ruled out. Three months after the operation, the patient’s symptoms regressed, the wrist motion was retained, and no signs of median nerve compression or recurrence were observed.

## Discussion

Rice bodies are a rare condition that was first reported in patients with tuberculous arthritis. Thereafter, rice bodies have been reported in cases with a wide variety of rheumatic and inflammatory diseases, including but not limited to rheumatoid arthritis, juvenile idiopathic arthritis, and lupus erythematosus. This condition is frequently observed in the following locations: subacromial bursa of the shoulder and the knee. However, the presence of rice bodies in the wrist is less common [1,3,4]. We carried out a thorough database search through PubMed (with the search query: “rice bod*” AND wrist) to identify all similar case reports in the literature (Tables 1, 2) [1,4-12].

**Table 1:** Presentation patterns of cases with rice bodies of the wrist in the literature YOP, year of publication; HIV, human immunodeficiency virus; NS, not significant; TB, tuberculosis; DM, diabetes mellitus; SLE, systemic lupus erythematosus; HTN, hypertension

Author (YOP)	Patient's characteristics		Location	Past medical history	Presentation			
	Age	Gender			Symptom/sign	Mass description	Laterality	Timing
Gillijns et al. (2022) [1]	90	-	Volar aspect	-	Painful swelling	-	Right	5 months
Sulaiman et al. (2016) [11]	71	Female	Dorsal part	HIV (since 2005)	Initial lesion: non-tender and mobile. Later, a new, red and tender lesion lateral to the initial lesion developed	1 cm	Right	3 years
Iyengar et al. (2011) [3]	72	Female	-	NS	Progressive painful swelling	8x6 cm	Bilateral	6 months
Woon et al, (2011) [12]	87	Male	Volar aspect	TB	Fluctuant mass	3x2 cm	Right	2 years
Woon et al, (2011) [12]	70	Male	Dorsum	TB - DM - HTN	Fluctuant mass	4 cm	Left	9 months
Woon et al, (2011) [12]	30	Female	Volar aspect	TB - SLE	Painless swelling	10 x 4 cm	Left	6 months
Woon et al, (2011) [12]	44	Male	-	TB - psoriasis	-	-	Left	5 years
Woon et al, (2011) [12]	-	-	-	TB	-	-	-	-
Woon et al, (2011) [12]	-	-	-	TB	-	-	-	-
Bayram et al. (2016) [13]	50	Male	Volar aspect	TB	Painful, red swelling	-	Right	2 years
Tyllianakis et al. (2006) [4]	61	Female	Volar aspect	NS	Mild pain and swelling	-	-	-
Celikyay et al. (2018) [5]	34	Male	Volar aspect	Penetrating injury to the wrist with foreign body removal	-	-	Right	-
Kurra et al. (2019) [10]	44	Female	Dorsum	TB	Painful mass	-	-	1 year
Korkmaz et al. (2021) [9]	42	Male	Volar aspect	TB	Slowly growing mass	-	Left	2 years
Ergun et al. (2008) [7]	32	Male	-	NS	Painless giant mass	-	Right	4 months
Chavan et al. (2012) [6]	57	Male	Dorsum	TB	Non-tender, non-compressible mobile swelling	-	Left	3 years
Hung et al. (2011) [14]	56	Female	-	NS	Painless-to-painful enlarging mass	-	Left	5 years

**Table 2:** Laboratory, radiological, and histopathological assessment of cases with rice bodies (of the wrist) and subsequent management outcomes YOP, year of publication; US, ultrasonography; MRI, magnetic resonance imaging; TB, tuberculosis; CRP, C-reactive protein; ESR, erythrocyte sedimentation rate; MTBC, mycobacterium tuberculosis complex; PCR, polymerase chain reaction; FU, follow-up; RF, rheumatoid factor; +, positive

Author (YOP)	Lab investigations			Radiological assessment		Management	Histopathology	Aspirate PCR	Outcome	FU
	CRP	ESR	Rheumatological assays	US	MRI					
Gillijns et al. (2022) [1]	Normal	Normal	Negative	Pronounced swelling of the synovial tissue of the flexor tendons	Extensive synovitis extending from the distal forearm into the hand with inclusions "rice bodies"	Synovectomy + carpal tunnel release + dissection of the mass	Lymphohistiocytic infiltrates consistent with rheumatoid nodules	-	Function: fully regained; pain: none	2 weeks
Sulaiman et al. (2016) [11]	-	-	-	-	Multiple rice bodies	Surgical excision of the mass and aspiration of the lesion	-	MTBC (+)	Lesion size subsided and healed	A few months
Iyengar et al. (2011) [3]	Normal	Elevated	Negative RF	Echogenic fluid on the palmar aspect of the wrist joint surrounding flexor tendons with intact neurovascular bundles and no bony erosion	-	Subtotal flexor tenosynovectomy	Several areas of fibrinoid necrosis, bounded by a layer of vaguely pallisaded histiocytes but no epitheloid granulomata or germinal centre	-	Recurrence: managed by revision surgery of the fibro-osseous canal. Complete resolution (at 1 year)	1 year
Woon et al. (2011) [12]	-	-	-	-	-	-	Necrotizing granulomatous infection	MTBC (+)	No recurrence	8 years
Woon et al. (2011) [12]	-	-	-	-	Carpal and metacarpal destruction with multiple abscesses	-	Necrotizing granulomatous inflammation with rare acid-fast bacilli	MTBC (+)	Healed satisfactorily. No recurrence	6 years
Woon et al. (2011) [12]	-	-	-	-	-	-	Tuberculoid granulomata with multinucleated giant cells	MTBC (+)	Nocturnal hand numbness. No recurrence	6 years
Woon et al. (2011) [12]	-	-	-	-	-	Debulking tenosynovectomy	Epithelioid granulomas with multinucleate giant cells	-	Wound healed. No recurrence	3 months
Woon et al. (2011) [12]	-	-	-	-	-	-	Epithelioid giant cell granulomas with central necrosis	MTBC (+)	No recurrence	1 month
Woon et al. (2011) [12]	-	-	-	-	-	-	Chronic granulomatous inflammation, epitheloid granulomas composed of epithelioid histiocytes, lymphocytes and multinucleated giant cells. Some granulomas contain central necrosis	-	No recurrence	4 years
Bayram et al. (2016) [13]	Normal	Normal	-	-	Millimetric and nodular images in flexor group tendon sheath	Surgical removal, and revision surgery (for recurrence)	-	-	Symptoms regressed. Recurrence (after six months)	6 months
Tyllianakis et al. (2006) [4]	Normal	Elevated	Negative	-	Acute tenosynovitis with an inflammatory mass inside the carpal tunnel	Surgical excision	-	-	Regained range of motion with no pain	1 year
Celikyay et al. (2018) [5]	-	-	-	-	Multiple nodules around the flexor tendons	-	Rice bodies and chronic granulomatous inflammation with caseous necrosis suggestive of TB synovitis	-	-	-
Kurra et al. (2019) [10]	-	-	Negative	Innumerable, smoothly marginated, isoechoic oval-shaped soft tissue bodies associated with the extensor tendon sheaths, measuring 0.5-1.0 cm	Innumerable nonenhancing, coffee-bean-shaped isointense bodies distending the third and fourth extensor tendon sheaths, in direct contact with the extensor tendons	Removal of the tendon sheath bodies and surgical exploration	-	Candida parapsilosis (+)	-	-
Korkmaz et al. (2021) [9]	Normal	Normal	-	-	Multiple rice bodies in the wrist and hand	Open biopsy	Granulomatous lesions with central necrosis	-	Symptoms resolved	4 months
Ergun et al. (2008) [7]	Normal	Slightly elevated	Negative	Noncalcified soft tissue mass over the wrist with normal joint and bony structures	A massive fluid collection involving the flexor tendons extending from the distal forearm into the carpal tunnel and the flexor tendons of the hand and fingers with normal underlying soft tissue	Surgical intervention (not specified)	-	-	Total recovery	3 months
Chavan et al. (2012) [6]	-	Normal	-	Giant cell tumor of extensor digitorum sheath	Extensor tendon sheath extraskeletal synovial Koch's, or giant cell tumor of tendon sheath	Surgical excision	Caseous necrosis with granuloma formation	-	-	-
Hung et al. (2011) [14]	Normal	Normal	-	-	-	Surgical excision	Multiple granulomas with focal central necrosis	MTBC (+)	No recurrence	1 year

In terms of clinical presentation, our case was quite similar to cases observed in the literature, highlighted by a mass that enlarges progressively with time. The presentation is acute (less than six months) [1,3,7] in some cases and chronic in others (more than six months) [6,9-12], and thus, some patients present with painful masses, while others present with painless masses. There is no trend in regard to laterality, as some cases present with masses in the right or left wrist [1,5,11-13]. These masses usually present on the dorsum of the wrist or on the volar aspect [1,5,9,10,12,13]. Laboratory investigations are usually non-specific, and therefore, they are minimally reported in the literature (Table 2).

Rice bodies usually originate, histologically, from the amorphous eosinophilic material (that is mainly composed of fibrin, fibronectin and collagen) in addition to polymorphonuclear cells [15-17]. This finding is quite similar to that of a rheumatoid nodule where, histologically, it is made up of a central area of fibrinoid necrosis with an outer layer of histiocytes covered peripherally by loose connective tissue. This is in line with our pathological findings, where the microscopic examination of rice bodies found in the right wrist of our case revealed nodules of clustered fibrin and polymorphonuclear cells with karyorrhexic debris, while the remaining fragments showed fibrous synovium rich in plasma and lymphoid aggregates, all of which is consistent with our patient’s history of rheumatoid arthritis. Important to mention, our patient presented initially with a painful swelling in the right wrist, and therefore, we considered initially the possibility of rheumatoid arthritis, gout, and giant cell tumor. Therefore, we carried out PD MRI to further assess this swelling, through which all findings were suggestive of “rice bodies”, consistent with the observations in the literature [1,11,12]. Based on previous results, rice bodies occur secondary to the inflammation of the synovial tissues, the formation of villi, and the proliferation and degeneration of the present synovium [15,17]. That being said, the exact mechanism through which rice bodies occur is yet to be confirmed.

The management of such cases differed widely in the literature, but all of them are focused on the removal of the mass either through subtotal synovectomy, debulking synovectomy, or aspiration [3,11,12]. The surgical excision of the mass usually results in satisfactory results in terms of regaining the function of the wrist, good wound healing, and resolution of presenting symptoms (particularly pain) [1,11,12]. Although rare, a few cases have reported recurrence following the initial surgery, and therefore, they underwent revision surgery [3,13]. In a previous report, the removal of rice bodies through lavage and aspiration has shown effectiveness in treating this condition, resulting in significant clinical improvement in the joint [17]. This therapeutic approach is considered feasible in large joints like the shoulder and knees, in which rice bodies are more frequent; however, performing this technique in small joints like the wrist can be challenging. In this context, synovectomy was proposed as an appropriate approach in such case [15]. The risk of rupture of the tendons of forearm flexors in tenosynovitis with lavage and aspiration makes synovectomy a better approach in terms of improvement in clinical outcomes with minimal safety risks [18]. In our case, rice bodies were removed through extensive debridement. Our case was followed up for three months after the removal of rice bodies, and showed no signs of recurrence. This is consistent with the literature since recurrence of rice bodies is a rare encounter and, to our knowledge, has been reported only once in a case with bilateral wrist flexor synovitis [3].

## Conclusions

Rice bodies in the wrist are a rare finding commonly associated with a chronic microinflammation of the synovium either in the context of rheumatoid arthritis or chronic infections. The diagnosis can be made through proper history, physical examination, and intensive radiological assessment. The management of such cases differs widely but focuses on the surgical removal of the mass, with satisfactory results obtained.
